# Early detection and severity classification of verticillium wilt in cotton stems using Raman spectroscopy and machine learning

**DOI:** 10.3389/fpls.2025.1649295

**Published:** 2025-10-01

**Authors:** Xuanzhang Wang, Jianan Chi, Xiao Zhang, Guangshuai Lu, Xuan Li, Chunli Wang, Lijun Wang, Nannan Zhang

**Affiliations:** ^1^ Country College of Information Engineering, Tarim University, Alar, China; ^2^ Key Laboratory of Tarim Oasis Agriculture (Tarim University), Ministry of Education, Alar, China; ^3^ Analysis and Testing Center, Tarim University, Alar, China

**Keywords:** Raman spectroscopy, cotton stems, verticillium wilt, disease severity classification, machine learning, CARS-INFO-SVM

## Abstract

The early detection of Verticillium wilt (VW) in cotton is a critical challenge in agricultural disease management. Cotton, a vital global textile resource, is severely threatened by this devastating disease. Traditional diagnostic methods, which often rely on manual expertise or destructive sampling, are limited by low efficiency and high subjectivity. In recent years, Raman spectroscopy has emerged as a promising solution due to its rapid, non-destructive, and highly sensitive characteristics for plant disease detection. In this study, we analyzed cotton stems using Raman spectroscopy, applying Savitzky-Golay (SG) smoothing combined with multiple preprocessing methods including Scaling and Shifting (SS), Standard Normal Variate (SNV), inverse first-order differential (1/SG)′, and multiplicative scatter correction (MSC). For baseline correction, we employed polynomial fitting (PolyFit) and adaptive iterative weighted penalized least squares (airPLS). Feature selection was performed using principal component analysis (PCA), successive projection algorithm (SPA), and competitive adaptive reweighted sampling (CARS).Three optimized models were developed: support vector machine (SVM) with weighted mean of vectors (INFO) algorithm, random forest (RF) enhanced by particle swarm optimization (PSO), and long short-term memory (LSTM) network optimized via chameleon swarm algorithm (CSA).The results show that the INFO-SVM model with SG-airPLS-(1/SG)′ -CARS preprocessing demonstrated superior performance, achieving 97.5% accuracy (0.974 F1-score) on training data and 90.0% accuracy (0.867 F1-score) on validation data, outperforming both PSO-RF and CSA-LSTM models. These results confirm that Raman spectroscopy integrated with optimized machine learning enables accurate VW classification in cotton stems. This method enables early disease detection during infection, facilitating timely fungicide application and reducing yield losses.

## 1 Introduction

Cotton (*Gossypium* spp.) is a vital global cash crop and a key raw material for the textile industry (Arya and Sarkar, 2024). As the world’s largest cotton producer and consumer, China plays a pivotal role in sustaining the global textile supply chain. Xinjiang Uygur Autonomous Region is the dominant cotton-producing area in China, contributing 83.2% of the country’s total cotton cultivation area in 2022 (National Bureau of Statistics of China, 2022). The region’s favorable natural conditions, including abundant sunlight and suitable soil, support high-quality cotton production, meeting both domestic and international demand.

However, cotton production faces significant challenges from pests and diseases, particularly Verticillium wilt (VW). In Xinjiang, nearly half of the major cotton-growing areas are affected by moderate-to-severe VW, with 24.1% classified as severely infected (Liu et al., 2015). This disease severely reduces cotton yield and quality, causing annual economic losses of 1.5 to 2 billion yuan in China (Cai et al., 2009; Wang, 2022). Early detection and effective management of VW are therefore critical to minimizing its impact on cotton production.

Cotton VW is a trans-regional disease characterized by widespread incidence, high prevalence, and a significant probability of occurrence (Liu et al., 2015). It is one of the most serious diseases affecting cotton production both in China and all over the world (Wang, 2012). *Verticillium dahlia* (*V. dahliae*) is a soil-borne fungus that primarily infects vascular bundle systems of cotton plants. After microsclerotia germinate in the soil, the mycelium can invade directly through cotton root hair cells, root epidermal cells, or root wounds (Bhandari et al., 2020; Zhu et al., 2023; Wu et al., 2025). It then penetrates the cortex and spreads throughout the plant via vascular conduits (Bolek et al., 2005). This infection mechanism complicates the early detection of VW, particularly during the seedling stage, because cotton plants typically do not exhibit obvious external symptoms. However, dissection examinations have revealed yellow-brown lesions in the xylem conduits of cotton stems (Palanga et al., 2021). As the disease progresses, cotton plants may show typical symptoms after the buds emerge, including green loss, yellowing, curling, and drying of leaf tissue between the main veins of the leaf blades (Zhang et al., 2025). In severe cases, this can lead to complete wilting and shedding of leaf blades (Yang et al., 2022). Owing to the absence of prominent external symptoms in the early stages of VW, its detection and identification pose significant challenges. Nevertheless, early diagnosis is essential to control the spread of the disease and to minimize economic losses. Consequently, the development of efficient and accurate early detection techniques has become a critical focus of current research on the management of cotton VW (Klosterman et al., 2009).

Currently, there are numerous detection methods for VW in cotton, however, traditional approaches have several disadvantages. The existing classification for early detection of cotton VW relies primarily on field identification and modern laboratory biochemical detection techniques (Jiang and Liu, 2002). Field identification typically necessitates manual assessment of diseased plants, which is not only time-consuming but also fails to accurately identify the disease during the incubation period (i.e., the early infection stage without typical symptoms). This limitation extends the timeframe for disease identification and creates conditions conducive to large-scale outbreaks of VW in cotton fields. While current laboratory-based methods like ELISA and PCR provide reliable pathogen identification, they exhibit fundamental limitations for early Verticillium wilt detection (Zhang et al., 2014). These techniques primarily target late-stage infection markers - ELISA detects pathogen-specific antibodies (Pérez-Artés et al., 2000; Yucel et al., 2005; Xu and Chen, 2000) and PCR identifies microbial DNA (Jiang and Liu, 2002) - both requiring substantial pathogen accumulation for accurate diagnosis. This inherent detection threshold means infections can only be confirmed after significant disease progression, missing the critical early intervention window. Consequently, these methods systematically miss the initial infection window when disease control measures would be most effective. These limitations have significantly hindered the rapid development of plant disease detection and control technologies. With advancements in technology, machine vision has been introduced in the realm of plant disease detection, significantly enhancing information collection efficiency and simplifying operational procedures (Lu et al., 2023). However, machine vision technology depends on the visible symptoms of the disease, and cannot capture the physiological responses of cotton plants during the early stages of infection (Mohanty et al., 2016). In contrast, hyperspectral imaging (HSI) technology, which captures both spatial and spectral information across hundreds of contiguous narrow bands, has emerged as a powerful tool for pre-symptomatic disease detection. Numerous studies have demonstrated its capability to identify subtle physiological and biochemical changes in cotton plants induced by *V. dahliae* infection, such as alterations in leaf temperature, chlorophyll fluorescence, and cellular structure (Lowe et al., 2017; Yang et al., 2024). However, its effectiveness in the very early, pre-symptomatic phase can be limited by the depth of tissue penetration and the relatively weak spectral signals associated with initial pathogen activity. Because cotton VW typically does not exhibit obvious external symptoms during the early stages of infection, both existing manual identification methods and machine vision techniques struggle to achieve accurate disease detection in this initial phase (Yang et al., 2022). Although traditional disease management strategies have reduced the spread and impact of this disease, they continue to face significant challenges in terms of early detection. Consequently, there is an urgent need to develop detection technologies capable of identifying VW during the pre-symptomatic phase, when early intervention can most effectively prevent disease establishment and spread.

Raman spectroscopy is a form of scattering spectroscopy that utilizes inelastic scattered light to identify the vibrational states of molecules (Schulz and Baranska, 2007). Each Raman peak in the spectrum corresponds to a specific molecular bond, allowing for molecular identification of analytes through the generation of a unique vibrational fingerprint (Saletnik et al., 2024). This technique is rapid, highly sensitive, and non-destructive, demonstrating significant potential in the field of plant disease detection (Negi and Anand, 2024). Raman spectroscopy can capture early physiological and biochemical changes associated with diseases by analyzing the molecular vibrational information of plant tissues, thereby facilitating early diagnosis (Juárez et al., 2023). In recent years, the application of Raman spectroscopy in agriculture has become an essential tool for disease detection owing to its advantages such as speed and environmental friendliness, leading to improved research outcomes. Tan et al. (2015) used Raman spectroscopy to analyze healthy and infected rice leaves affected by rice blast disease, enabling early detection in cold regions. Whereas Zhang (2016) used spectral and spectral imaging techniques to detect Sclerotinia stem rot in oilseed rape leaves, Farber et al. (2019b) applied Raman spectroscopy to identify rose rosette infection in rose plants. Farber Charles et al. used Raman spectroscopy to identify rose rosette infection (Farber et al., 2019b), and Farber Charles et al. detected wheat streak mosaic virus and barley yellow dwarf virus in wheat (Farber et al., 2020). Mandri et al. employed Raman spectroscopy to identify tomato plants infected with Tomato yellow leaf curl Sardinia virus and Tomato spotted wilt virus (Mandrile et al., 2019). Sanchez Lee et al. employed Raman spectroscopy for early detection and confirmatory diagnosis of Xanthomonas-induced diseases in citrus and grapefruit trees, demonstrating its potential as a sensitive alternative to qPCR-based pathogen detection (Sanchez et al., 2020). Vallejo-Perez et al. (2016), employed a portable Raman spectrometer to acquire spectral signatures from healthy, latent, and ‘Candidatus’ *Liberibacter asiaticus*-infected citrus leaves, utilizing PCA-LDA for spectral differentiation with 89% diagnostic accuracy. These studies demonstrate that Raman spectroscopy can identify crop-specific components, such as carbohydrates, amino acids, proteins, and lipids, through spectral peak analysis (Schulz and Baranska, 2007; Saletnik et al., 2024). They also highlight its significant potential for the identification and differentiation of early crop diseases by detecting pathogen-specific changes in plant metabolism (Tan et al., 2015; Zhang, 2016; Farber et al., 2019b, 2020; Mandrile et al., 2019; Sanchez et al., 2020). Furthermore, the use of portable Raman spectrometers enhances the practicality of Raman spectroscopy, enabling its direct application in the confirmatory diagnosis of viral diseases with high diagnostic accuracy (Vallejo-Perez et al., 2016; Sanchez et al., 2020). The objective of this study was to establish a rapid and accurate detection model for Verticillium wilt in cotton stems that could differentiate between varying degrees of cotton stem Verticillium wilt. This study aimed to provide an efficient and reliable technical tool for early diagnosis, precise prevention, and control of cotton stems VW. In practical applications, this technology can assist cotton farmers in the timely detection of issues during the early stages of a disease, enabling them to implement targeted preventive and control measures. These measures may include the rational application of fungicides and adjustments to planting strategies, which can effectively mitigate the spread of the disease, reduce yield loss, and safeguard the quality of cotton fiber. This is of great significance for promoting sustainable development of the cotton industry and enhancing the economic and social benefits of cotton production. Furthermore, this study offers new ideas and methods for the detection and diagnosis of other plant diseases, thereby advancing the application of spectral technology and machine learning in the agricultural sector.

## 2 Materials and methods

**Figure 1:**
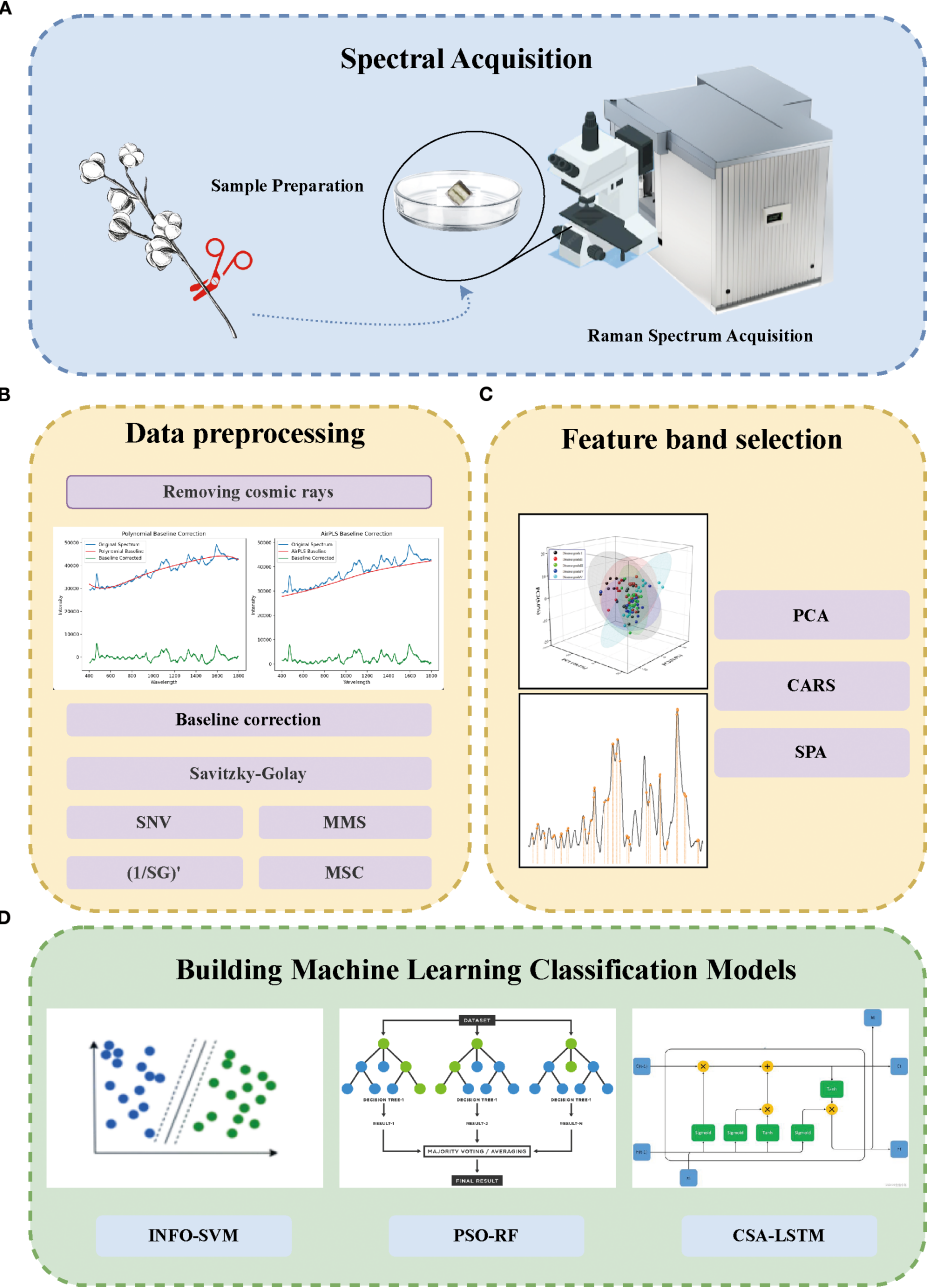
Methodological scheme for the rapid grading of cotton stalk Verticillium wilt (VW) disease using Raman spectroscopy and machine learning. **(A)** Sample Processing and Raman Spectrum Acquisition: Cotton stalks were collected, preprocessed, and their Raman spectra were acquired using Raman confocal microscopy. **(B)** Data Preprocessing: Spectral data were processed by removing cosmic rays, performing baseline correction, and applying smoothing techniques. **(C)** Feature Selection: Principal Component Analysis (PCA), Competitive Adaptive Reweighting Sampling (CARS), and Successive Projection Algorithm (SPA) were applied to select key spectral features from the preprocessed data. **(D)** Machine Learning Model Development: Classification models, including Weighted Mean of Vectors-Support Vector Machine (INFO-SVM), Particle Swarm Optimization-Random Forest (PSO-RF), and Chameleon Optimization Algorithm-Long Short-Term Memory (CSA-LSTM), were constructed to evaluate the classification performance of Raman spectra for cotton stalks with varying VW disease severity levels.

To establish a rapid and accurate detection model for cotton stem blight and distinguish different degrees of cotton stem blight, this study adopted the technical process shown in **Figure 1**. First, in the spectral acquisition stage (**Figure 1A**), cotton samples were prepared and their Raman spectra were collected. Then, in the data preprocessing stage (**Figure 1B**), steps such as removing cosmic rays, baseline correction, and applying algorithms like SG, SNV, MMS, (1/SG)’, and MSC were carried out. Next, in the feature band selection stage (**Figure 1C**), methods including PCA, CARS, and SPA were utilized. Finally, in the stage of building machine learning classification models (**Figure 1D**), models such as INFO-SVM, PSO-RF, and CSA-LSTM were constructed for classification.

### 2.1 Test sample collection

**Figure 2:**
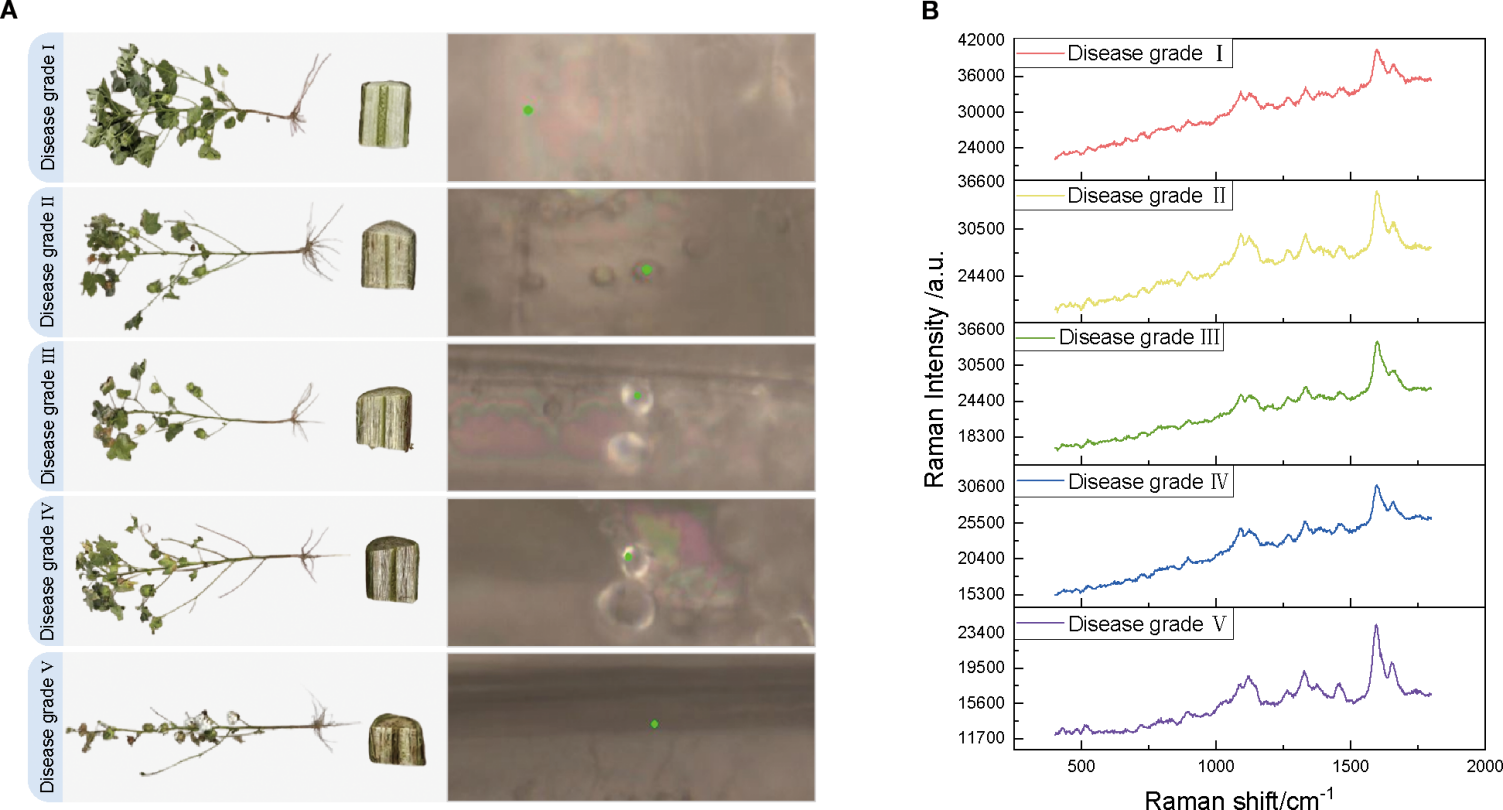
Comparison of cotton VW appearance, stem characteristics, and raw Raman spectra for different disease classes. **(A)** Schematic diagram of the appearance of cotton plants with VW disease classes I–V, stem cross-section, and selected points (marked by green dots) irradiated by Raman spectra under a microscope. **(B)** Original Raman spectra corresponding to the five VW disease classes.

In this controlled study, the experimental samples were selected from Tahe 2 cotton plants grown in pots in sterilized field soil (pH 8.2) under the conditions of the cotton growing greenhouse at Tarim University (day/night temperature difference of 28/22 °C, relative humidity of 60%, and a photoperiod of 14 h) and inoculated at the four-leaf stage with five different concentrations of *Verticillium dahliae* strains by root dipping Vd080 conidia (1 × 10³, 1 × 10^4^, 1 × 10^5^, 1 × 10^6^, and 1 × 10^7^ conidia/mL, courtesy of the Plant Pathology Laboratory, Tarim University) for simulating early infestation. Disease severity was categorized into five classes based on symptom development at 21 days post-inoculation (dpi): class I (0%, no symptoms), class II (vascular browning ≤ 25%), class III (25-50%), class IV (50-75%), and class V (≥75%, severe wilting), as shown in **Figure 2A**. **Figure 2A** illustrates representative images of cotton stems for each disease severity class, highlighting the progression of vascular browning and wilting across the five grades. Twenty biological replicates were performed at a time. These replicates consisted of 20 independent experiments, each with a separate set of cotton plants grown and inoculated under identical conditions. Stem segments (10 cm long) were collected 3 cm above the soil level, carefully avoiding epidermal and pith tissue. The consistency of the tissue sections was strictly controlled during sampling to minimize background interference. Ten vascular regions from each sample were analyzed using Raman spectroscopy within 24 hours of collection (stored at 4°C) to detect concentration-dependent pathological changes. Previous studies have confirmed that storing plant tissue samples at 4°C for up to 24 hours does not significantly alter Raman spectra, ensuring the reliability of the spectral data (Schulz and Baranska, 2007; Sanchez et al., 2020).

### 2.2 Raman spectroscopy data acquisition

The spectral data acquisition for this study was conducted at the Analysis and Testing Center of Tarim University using a HORIBA LabRAM Soleil-type Raman microscope (France) equipped with a 532 nm laser light source to minimize fluorescence background interference. The experimental parameters were established as follows: 42 mW laser power (to prevent thermal damage to the samples), a 20-second integration time (to enhance the signal-to-noise ratio [SNR]), 600 nm grating, and 50× objective lens (NA = 0.75). The spectra spanned a range of 400–1800 cm-¹ with a resolution of 1.15 cm-1. The samples were categorized into five grades based on disease severity (Grades I–V) determined by the percentage of lesions on the stem surface, which ranged from 0% to ≥75%. For each group, 20 cotton stems were selected, and 0.5–1 cm transverse slices of the stem segments were prepared by cutting the roots at 0–3 cm. These slices were then washed and dried in sterile deionized water, and the vascular bundles were mounted on slides with the cut surface facing upwards. To minimize the effects of tissue heterogeneity, 10 sites within a 1 mm² area were randomly selected in the xylem region of each sample for spectral acquisition, and the average value was recorded as the Raman spectrum of the sample (**Figure 2B**). **Figure 2B** shows representative Raman spectra for each disease severity grade, with key spectral peaks indicating molecular changes associated with *Verticillium dahliae* infection across the five classes. In total, 100 spectral data points were obtained. The wavelength was calibrated in silico (520.7 cm-1 peak) throughout the experiment, with calibration performed daily before each experimental session to ensure spectral accuracy. Ambient humidity was maintained at 40–50%, and the stability of the instrument was verified (with laser power fluctuations <2% and a repeat spectral correlation coefficient >0.98) to ensure that the data were reproducible and correlated with the pathological features.

### 2.3 Data preprocessing and feature selection

#### 2.3.1 Preprocessing methods

Lignin in cotton stems contains aromatic ring structures that tend to produce a strong fluorescent background under laser excitation (Laehdetie et al., 2013). In addition, metabolites of organic matter such as cellulose and hemicellulose introduce interference signals into the spectrum. These interferences mask the Raman signals, leading to a spectral baseline drift and increased noise (Zhang et al., 2024). To address these issues when measuring the Raman spectra of cotton stems, the built-in algorithm of the LabSpec6 software was initially used to automatically identify and interpolate high-intensity transient spikes triggered by cosmic rays (Cappel et al., 2010). This step helps mitigate the anomalous signal interference caused by high-energy particles (Ehrentreich and Sümmchen, 2001). To address the baseline drift caused by the strong fluorescence background of the cotton stem tissue, this study compared two baseline correction strategies: polynomial fitting (PolyFit) and airPLS. The former utilizes low-order polynomials to simulate the fluorescence trend but is vulnerable to interference from complex spectral regions (Zhao et al., 2007). In contrast, the latter uses asymmetrically weighted iterative optimization for dynamic baseline fitting, allowing it to better adapt to the heterogeneous fluorescence characteristics of plant tissues (Zhang et al., 2010). This approach effectively reduces interference, thereby enhancing the quality of the spectral data and improving the accuracy of subsequent analyses (Liu et al., 2019; Zhang et al., 2010).

Photon noise was also suppressed using Savitzky-Golay (SG) processing to enhance the SNR while preserving the characteristic peak morphology (Clupek et al., 2007). The optical range difference was eliminated through scaling and shifting (SS), the effects of scattering were compensated using a standard normal variate (SNV), and the effect of surface roughness was addressed using multiplicative scatter correction (MSC) (Afseth et al., 2006; Dhanoa and Lister, 1994; Kachrimanis et al., 2007). Inverse first-order differentiation (1/SG)′ was introduced to improve the resolution of weak peaks. All algorithms were implemented on the Python 3.10 platform using the sci-kit-learn library, which provides a solid foundation for high-quality spectral data for the subsequent hierarchical modeling of VW.

#### 2.3.2 Spectral characterization band selection

PCA, SPA, and CARS were used in this study to address the redundancy inherent in high-dimensional data and enhance the extraction of biochemical features specific to VW. PCA identifies the principal components that capture the maximum variance through an orthogonal transformation. However, this process may diminish the local nonlinear responses associated with disease classification while compressing the data dimensionality (Robert et al., 2023; Tariq et al., 2024). SPA iteratively selects feature wavelengths based on the least covariance criterion, and its greedy search strategy enhances model interpretability, although it may lack sensitivity to synergistic effects among discrete bands (Balabin and Smirnov, 2011; Ma et al., 2024). By contrast, CARS integrates spectral features with disease phenotypic correlations using Monte Carlo sampling and dynamic weighting mechanisms. To improve the stability, the randomness of variable screening should be enhanced through repeated calculations (Li et al., 2014). The application of these three algorithms provides a robust spectroscopic foundation for the development of an effective disease classification model using multidimensional feature fusion.

After preprocessing, the Raman spectral data were stratified and randomly divided into a training set and a test set at an 80:20 ratio to construct the classification model.

### 2.4 Model construction methods

#### 2.4.1 INFO-SVM modeling

**Figure 3:**
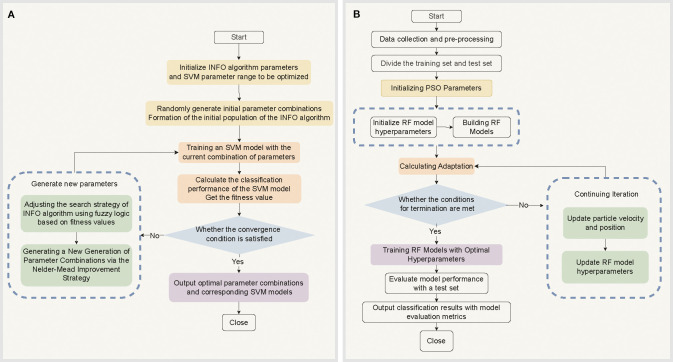
Optimization Algorithm Flowchart. **(A)** INFO algorithm-optimized SVM model parameters and classification process. **(B)** RF model training, evaluation, and classification process based on PSO-optimized hyperparameters.


A support vector machine (SVM) is a machine-learning algorithm grounded in a robust theoretical framework known for its exceptional classification performance. The effectiveness of SVMs is significantly influenced by the selection of the kernel function parameters γ and penalty coefficient C. However, optimizing these parameters often leads to local optima and incurs high computational costs (Li et al., 2015). To address these challenges, this study introduces an weighted mean of vectors (INFO) algorithm. The primary objective of the INFO-SVM method is to minimize the classification error while maximizing the generalization performance of the model within a complex parameter space by optimizing the kernel function parameters and penalty coefficients of the SVM (Ahmadianfar et al., 2022; Wan et al., 2024). By integrating an enhanced Nelder-Mead method with a fuzzy optimization strategy, the search step size is dynamically adjusted, and fuzzy logic is used to manage the uncertain parameters. This approach aims to minimize the classification error and maximize the generalization performance of the model while efficiently searching for an optimal solution in an intricate parameter space (Ahmadianfar et al., 2022; Wan et al., 2024). The core process in **Figure 3A**: in which 80% of the preprocessed Raman spectral data from cotton stems were used as the training set ${\left\{(x_{\dot{i}},y_{i})\right\}}_{i=1}^{n}$ , where x_i_ is the Raman spectral feature vector of cotton stems, and y_i_ is the corresponding classification label for cotton stem Verticillium wilt. The goal of SVM is to identify the optimal classification hyperplane *W* ^T^ ϕ(x)+b=0, where ϕ(x) is the kernel function mapping, *W* is the weight vector, and *b* is the bias term. The optimization problem can be expressed as (Equation 1)

(1) $$\underset{W,b,\xi }{min}\frac{1}{2}∥W{∥}^{2}+C\sum_{\text{i}=1}^{n}\xi_{\text{i}}$$

The constraints are (Equation 2):


(2) $${\text{y}}_{\text{i}}(W^{T}\phi ({\text{x}}_{\dot{i}})+b\geq 1-\xi_{\text{i}},\;\xi_{\text{i}}\geq 0,\text{ i}=1,\ldots ,n$$

where ξ_i_ are the slack variables and *C* is the penalty coefficient. The INFO algorithm evaluates the model performance by dynamically optimizing the kernel function parameters *γ* and penalty coefficients *C* using the fitness function f (γ, *C*) (Equation 3):


(3) f(γ,C)=Accuracy(γ,C)−λ·Generalization Error(γ,C)

Where λ is a trade-off factor. The INFO algorithm iteratively updates the parameter combinations (γ, C) using the Nelder-Mead method until the fitness function converges to the global local optimal solution (Equation 4).


(4) $$\left(\gamma^{*},C^{*})=\arg\underset{\gamma ,C}{max}f(\gamma ,C\right)$$

Ultimately, the SVM model is trained using optimal parameters (γ*, *C* *), which significantly enhance the classification accuracy and generalization performance while simultaneously mitigating the risk of falling into a local optimum, which is a common issue with traditional methods. Its efficient parameter-search strategy reduces computational costs, and when combined with fuzzy logic, improves the adaptability of the model to complex data distributions. This approach offers an effective and robust solution for the classification of cotton stem Verticillium wilt (Cortes and Vapnik, 1995; Ahmadianfar et al., 2022; Wan et al., 2024).

#### 2.4.2 PSO-RF modeling

The PSO-RF model offers an efficient and robust solution for classifying cotton stem Verticillium wilt by integrating particle swarm optimization (PSO) with random forest (RF) algorithms (Chatrsimab et al., 2020). As an ensemble method, RF demonstrates exceptional classification performance when handling high-dimensional, nonlinear Raman spectral data by constructing multiple decision trees and aggregating their predictions (Khan et al., 2017). However, the effectiveness of the RF is significantly influenced by the selection of hyperparameters (e.g., the number of trees, maximum depth, and minimum number of samples for splits). Traditional optimization methods, such as grid search, struggle to quickly identify optimal parameter combinations in complex data scenarios because of their high computational cost and low efficiency. Consequently, PSO algorithms are introduced to address these challenges. The PSO algorithm is based on the principle of swarm intelligence optimization (Bergstra and Bengio, 2012; Chatrsimab et al., 2020). It efficiently explores the hyperparameter space and approximates the global optimal solution by simulating the dynamic updates of particle positions and speed adjustments within the search space as well as a mechanism for sharing information about both global and local optimal solutions (Wu et al., 2023). The workflow of the PSO-RF algorithm is divided into two main stages. In the first stage, the RF hyperparameters are optimized using the PSO algorithm, which searches for the optimal parameter combinations in the hyperparameter space through continuous iterations. In the second stage, the optimized hyperparameters are applied to the RF model. The RF model is trained using the training data, and the trained model is subsequently used to classify and predict unknown data (Chatrsimab et al., 2020; Xiao et al., 2022). The specific process in **Figure 3B**: First, the position vector x_i_ of each particle in the swarm is defined and the particle velocity *V_i_* is initialized. The fitness function f(x_i_) (Equation 5), typically defined as the classification accuracy, is used to evaluate the performance of the RF model, as follows (Equation 6):


(5) $$x_{i}=(n_{trees},d_{max},s_{min})$$

(6) $$f(x_{i})=Accuracy(x_{i})$$

where the hyperparameters of RF, n_trees_, d_max_, and s_min_, denote the number of trees, the maximum depth, and the minimum number of sample splits, respectively. The particles update their speed and position according to the individual historical optimal position P_i_ and the global optimal position *g* (Equations 7, 8).


(7) $$V_{i}(t+1)=\omega \cdot V_{i}(t)+c_{1}\cdot r_{1}\cdot (P_{i}-x_{i}(t))+c_{2}\cdot r_{2}\cdot (g-x_{i}(t))$$

(8) $$x_{i}(t+1)=x_{i}(t)+V_{i}(t+1)$$

where ω is the inertia weight, c_1_ and c_2_ are the learning factors, r_1_ and r_2_ are random numbers. The optimal hyperparameter combination x* is gradually approximated by iteratively updating the particle positions. Ultimately, the RF model is trained using x*, and its classification performance is evaluated on a test set to achieve an accurate classification of cotton stem Verticillium wilt. Applying PSO to the hyperparameter optimization of RF not only significantly enhances the accuracy and generalization capabilities of the model for grading cotton stem Verticillium wilt but also dramatically reduces computational costs. In addition, it adapts well to various data distributions and disease grading scenarios, demonstrating exceptional generalization ability. This approach provides reliable technical support for early diagnosis of the disease and precise prevention and control.

#### 2.4.3 CSA-LSTM modeling

Long short-term memory (LSTM) is a deep learning model that effectively processes time-series data and addresses the issues of gradient vanishing and gradient explosion that are commonly encountered in traditional recurrent neural networks. This is achieved by introducing a gating mechanism that enables the retention of long-term information (Zhang et al., 2018). However, the performance of LSTM is highly dependent on the selection of the hyperparameters. Traditional optimization methods, such as grid search and stochastic search, are often inefficient and susceptible to local optima, which can hinder improvements in the model performance (Bergstra and Bengio, 2012). Chameleon swarm algorithm (CSA) mimics the predatory behavior of chameleons and exhibits strong global search capabilities and rapid convergence through a unique visual perception and fast localization mechanism, effectively mitigating the local optimum problem (Braik, 2021). The core of the CSA-LSTM algorithm is to use CSA to optimize the hyperparameters of LSTM networks, thereby enhancing the classification performance of LSTM in grading cotton stem Verticillium wilt (Abba et al., 2023). The workflow is as follows. First, the chameleon population is randomly initialized, with the position vector of each chameleon representing a set of hyperparameter combinations for the LSTM. Subsequently, these hyperparameter combinations are applied to the LSTM models and the performance of each model is evaluated using a defined fitness function. The predatory behavior of the chameleon is simulated in three phases: searching for, locating, and capturing prey. During this process, the position of the chameleon is continuously adjusted within the search space, allowing the optimization of the LSTM hyperparameters. After numerous iterations, the optimal combination of hyperparameters is selected to initialize the LSTM model (Al Bataineh and Kaur, 2021) once the preset termination conditions are met. Ultimately, the optimized LSTM model was used to classify the cotton stems VW data to achieve accurate disease classification. The integration of CSA into LSTM hyperparameter optimization is anticipated to significantly enhance model performance and provide efficient and reliable technical support for practical applications such as the classification of cotton stem Verticillium wilt.

### 2.5 Model evaluation indicators

In this study, accuracy (Equation 9) and F1-score (Equation 10) were used as the primary evaluation metrics to quantify the overall performance of the model in grading the severity of cotton stem Verticillium wilt. The accuracy reflects the overall classification accuracy of the model and is suitable for assessing its diagnostic efficacy, such as distinguishing between different levels of VW infection. However, its sensitivity to class imbalance may lead to an overestimation of the predictive advantage of majority classes (Mandrile et al., 2019). To address this limitation, we introduced the F1-score, which harmonizes the means of precision and recall. This approach emphasizes the risk of detecting early stage diseases in field samples due to hidden symptoms, aligning with the fundamental requirements of “early diagnosis and early intervention” in plant pathology (Barbedo, 2019).


(9) Accuracy=TP+TN/(TP+TN+FP+FN)

(10) F1−Score=2·(Precision·Recall)/(Precision+Recall)

where Precision is the precision rate and Recall is the recall rate.

The advantages of combining these two metrics are particularly significant in the grading scenario for VW. The accuracy metric validates the model’s consistent identification of dominant symptoms, such as vascular browning, whereas the F1-score enhances the sensitivity to subtle spectral features present during the initial infection stage, thereby mitigating the model bias caused by a skewed sample distribution (Vallejo-Perez et al., 2016). The experimental component further confirmed the robustness of the index through standard deviation analysis using ten-fold cross-validation. In addition, it addressed the cross-grade misclassification pattern by integrating the confusion matrix, which provided a foundation for optimizing the grading thresholds. This further demonstrates the capability of the evaluation system to characterize the dynamic pathological mechanisms of VW.

## 3 Results

### 3.1 Spectral preprocessing

**Figure 4:**
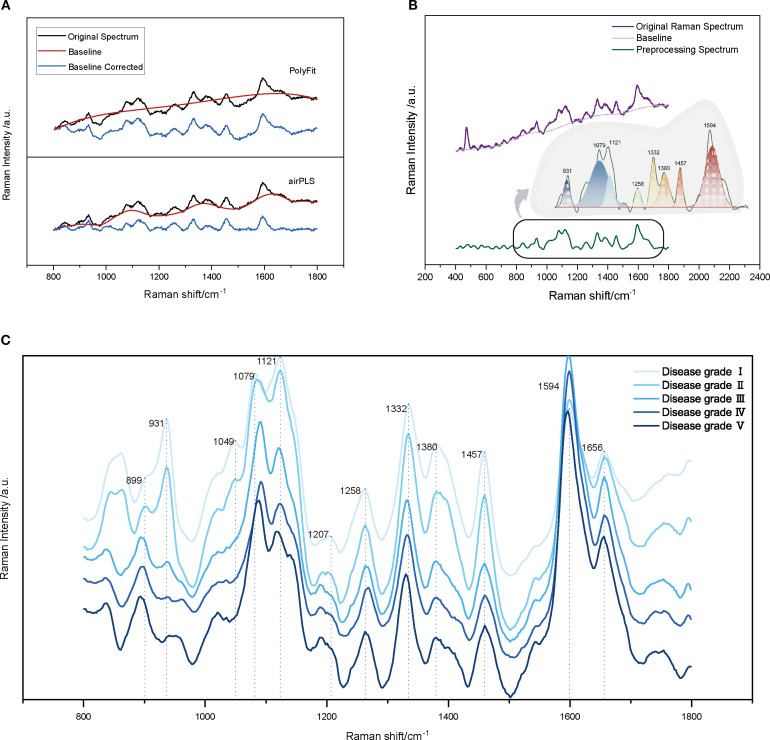
Pre-processing and Spectral Analysis of Raman Spectroscopy for Cotton Stems. **(A)** Comparison of baseline correction algorithms: PolyFit and airPLS, with the black line representing the original spectrum, the red line showing the estimated baseline, and the blue line indicating the corrected spectrum. **(B)** Raman spectrum with characteristic peaks after baseline correction and smoothing. **(C)** Comparison of average Raman spectra for cotton stems across Verticillium dahliae disease severity grades I-V (0%, ≤25%, 25-50%, 50-75%, and ≥75% vascular browning or wilting), with each line representing the average spectrum of 20 stem samples per grade.

The parameters for each method were optimized during the preprocessing stage. For the SG processing, the number of smoothing points was set to eight to effectively denoise the data while preserving the primary features of the spectrum. In the baseline correction step, this study compared two commonly used methods, PolyFit and airPLS. The PolyFit method estimates the baseline by fitting a low-order polynomial with a chosen order of three (Gan et al., 2006; Lieber and Mahadevan-Jansen, 2003). This choice strikes a balance between the fitting accuracy and the risk of overfitting. Although this method is computationally simple and easy to implement, it may lack the flexibility required to address the complex baselines (Gan et al., 2006). In contrast, the airPLS method optimizes the number of iterations and penalty weights through cross-validation, allowing better management of the nonlinear baseline drift and complex fluorescence backgrounds (Zhang et al., 2010). In addition, SS and SNV transformations were used to eliminate discrepancies in the spectral intensity, whereas (1/SG)′ was applied to enhance subtle features within the spectra. **Figure 4A** presents a comparison between the original and Raman spectra after baseline correction. As illustrated, airPLS baseline correction significantly suppressed the background fluorescence and noise signals in the spectra. The intensity range of the Raman peaks became more concentrated, the baseline fitting curve aligned more closely with the low-frequency portion of the original spectra, and the main feature peaks were more distinctly visible, allowing for better capture of the complex baseline variations. However, the polynomial-fit-corrected spectra exhibited overfitting or underfitting in certain regions, which led to distortions in the intensities or shapes of the characteristic peaks. This indicates that airPLS is more effective in managing complex fluorescence backgrounds, and its ability to remove fluorescence backgrounds surpasses that of PolyFit, thereby better preserving the spectral information related to VW.

### 3.2 Raman peak resolution

The Raman fingerprint region (300–1800 cm^-^¹) provides crucial information regarding the biochemical composition of cotton stems, as the vibrational bands within this range are closely associated with structural polymers such as lignin, cellulose, and hemicellulose (Agarwal, 2006; Gierlinger and Schwanninger, 2007; Smith and Dent, 2005). We first analyzed the Raman spectra of the VW-infected cotton stems (**Figure 4B**). **Table 1** summarizes the characteristic vibrational bands and their corresponding biochemical assignments. Notably, the peak intensities at 931, 1332, 1457, and 1594 cm^-^¹ reflect the degree of lignin polymerization, a key structural polymer that dominates the Raman spectral features of cotton stems. The Raman peak at 931 cm^-^¹ corresponded to the vibrational mode associated with C–C–H. When *V. dahliae* infects cotton stems, it disrupts the normal physiological metabolism of plants, causing an imbalance between the synthesis and decomposition of intracellular substances, particularly affecting polysaccharide metabolism (Shaban et al., 2018; Xiong et al., 2021). This disruption affects the metabolism of polysaccharides and alters the chemical environment in which chemical bonds, such as C–C–H, are embedded, leading to shifts in their vibrational frequencies (Egging et al., 2018; Gierlinger and Schwanninger, 2007). 1332 cm^-^¹ is attributed to -CH deformation and -CCH bending, which may result from the degradation of lignin. This degradation could lead to the breaking or deformation of the -CH bond, indicating potential changes in the lignin structure of cotton stems after Verticillium wilt infection. Such alterations may affect the physical and chemical properties of stems. The peak at 1457 cm^-^¹ corresponds to the bending of CH_3_ in OCH_3_. In addition, the strong aromatic ring symmetry stretching vibration observed at 1594 cm^-^¹ further confirms the presence of lignin, suggesting that the aryl-related chemical bonds and functional groups within the cotton stems were affected during the infection process with VW disease, potentially altering their internal lignin structure. Cellulose vibrations were prominent at 1079, 1121, and 1380 cm^-^¹. The Raman peak at 1079 cm^-^¹ can be attributed to the vibrations of the C-O-C or C-C bonds. In contrast, the peak at 1121 cm^-^¹was assigned to symmetric stretching of the glycosidic C-O-C bond. The Raman features appearing at 1380 cm^-^¹ were associated with -CCH, -CHO, and -COH bond bending. Hemicellulose, likely in the form of xyloglucan, contributes to the -CH and -COH bending observed in the 1258 cm^-^¹ band, consistent with its mixed polysaccharide structure.

**Table 1:** Raman band attribution in VW cotton stems.

Band (cm^−1^)	Vibrational assignment	Polymer
931	CCH Vibration	Lignin (Yang et al., 2023)
1079	C-O-C or C-C bond vibration	Cellulose (Agarwal and Ralph, 1997)
1121	-COC-symmetric stretching (glycosides)	Cellulose (Gorzsás, 2017)
1258	-CH, -COH bend	Hemicellulose (Gorzsás, 2017)
1332	- CH deformation and - CCH bending	Lignin (Gorzsás, 2017)
1380	- CCH, - CHO, - COH and C-O bending	Lignin (Gorzsás, 2017); Fusaric acid (Rosado et al., 2016)
1457	CH3 bends in OCH3	Cellulose (Yang et al., 2023)
1594	Aryl ring symmetric stretching vibrations	Lignin (Cao et al., 2006)

The Raman spectra of the cotton stem exhibited significant changes with increasing levels of VW infestation (**Figure 4C**). The primary differences between the early and late spectra were observed at 899, 931, and 1594 cm^-^¹. Notably, the Raman characteristic peak at 1594 cm^-^¹ exhibited a higher intensity during the middle stage of VW infestation (disease grades II–III) than during the early stage. This increase is attributed to the activation of plant defense mechanisms in response to the initial infestation (Pomar et al., 2004). In the early stages, the plant enhances the mechanical strength of its cell wall by increasing lignin synthesis, which promotes the deposition of lignin around vascular bundles, thereby strengthening the mechanical barrier (Klopfenstein et al., 1991; Pomar et al., 2004). However, as the disease progresses, plant defense mechanisms may gradually weaken, allowing the pathogen to secrete various cell wall-degrading enzymes, including lignin-degrading enzymes. These enzymes can disrupt the structure of lignin, resulting in decreased capacity for lignin synthesis and a reduction in its overall content (Yucel et al., 2005; Pomar et al., 2004). This phenomenon accounts for the observed decrease in the intensity of the Raman characteristic peaks at 931 and 1594 cm^-^¹ during the later stages of the disease. Disruption of the cellulose structure was evidenced by an increase in the half-height width of the peak at 1079 cm^-^¹, indicating a reduction in crystallinity due to the breakage of β-1,4-glycosidic bonds. This phenomenon further confirmed the degradation of cell walls by cellulases secreted by pathogenic fungi. Additionally, the Raman peak at 899 cm^-^¹ was correlated with the ν(CCH), ν(COH) vibrational mode of pectin. As the severity of VW intensified, pectin continued to degrade, resulting in a shift of the characteristic peaks to lower wavenumbers (~850 cm^-^¹), whereas the intensity of Raman peaks related to pectin (e.g., 850–900 cm^-^¹) decreased significantly. This alteration reflects the degradation of pectin under the influence of pectinase secreted by the pathogenic fungi. The observed changes in the spectral features indicate the degradation and structural alteration of the primary components of the cotton stem cell wall (lignin, cellulose, and pectin) during infestation by *Verticillium dahliae* (Schulz and Baranska, 2007; Agarwal, 2006). These hierarchical changes in biochemical characteristics provide a molecular spectroscopic foundation for analyzing the pathogenic mechanisms of VW and breeding disease-resistant varieties. This study is of great significance for the in-depth study of microstructural changes in cotton stems after VW infection, and the development of effective disease detection and control methods.

### 3.3 Characteristic band selection

**Figure 5:**
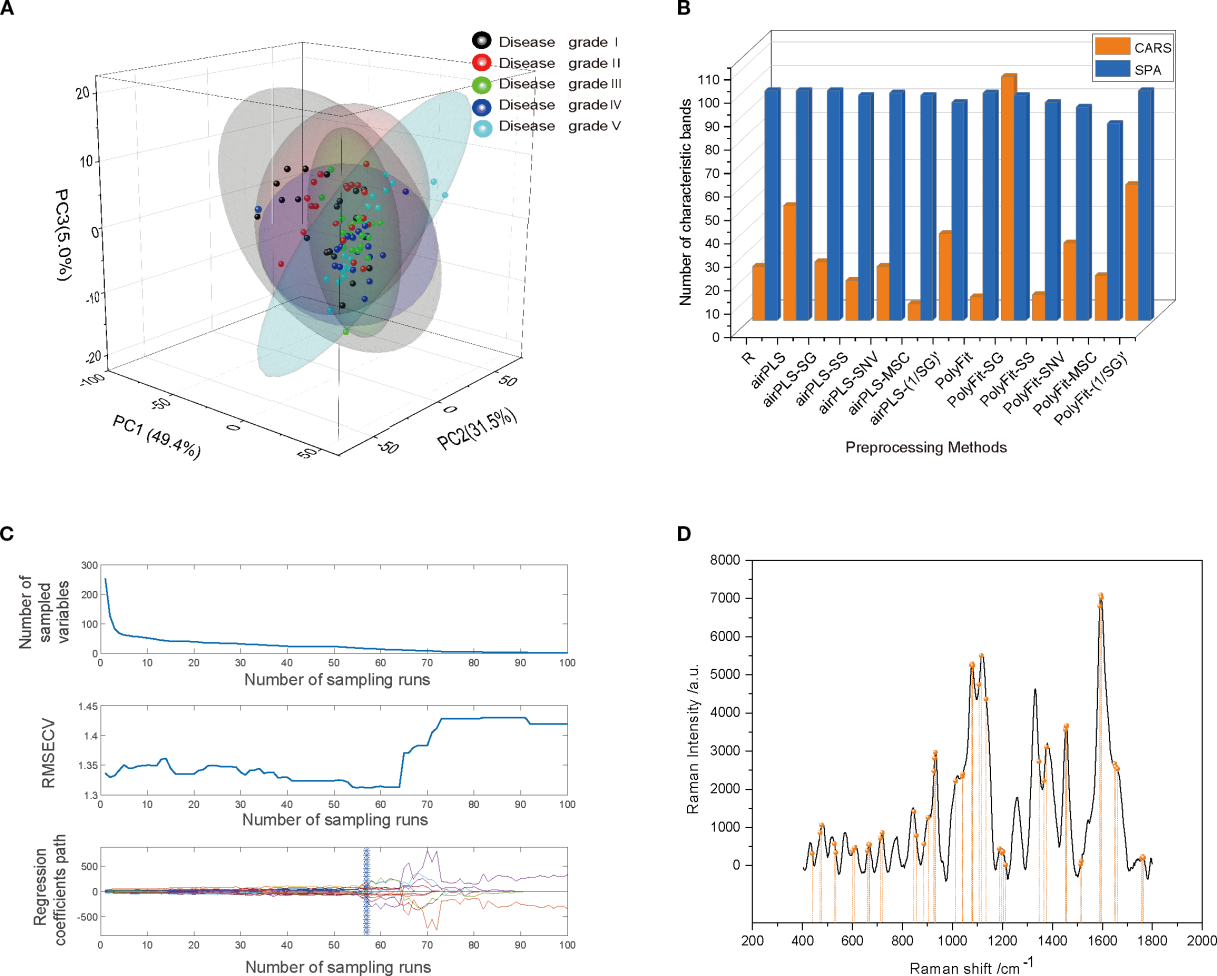
**(A)** PCA plot of model scores. **(B)** Number of feature bands resulting from different preprocessing steps for the SPA and CARS feature band selection algorithms. **(C)** CARS process for spectral feature band selection in the INFO-SVM classification model. The number of sampling runs was 100, and the RMSECV showed a decreasing and then an increasing trend. The optimal set of feature wavelengths was selected when the number of sampling runs was 57. **(D)** Spectral feature band selection by CARS in PSO-RF classification model.

Before constructing the classification model for cotton stem Verticillium wilt, preprocessed Raman spectral data were analyzed using PCA to preliminarily assess the spectral distribution characteristics of different grades of cotton stem Verticillium wilt infection (disease grades I–V) through downscaling and visualization. A total of 100 cotton stem Raman spectral data points were inputted into the PCA model. The results (**Figure 5A**) indicated that the first two principal components (PC1 and PC2) accounted for 49.4% and 31.5% of the variance, respectively, yielding a cumulative contribution of 80.9%. The cumulative contribution of the first three PCs reached 85.9%, suggesting that these components effectively captured the primary features of the spectral data. However, as illustrated in the PCA score plot, although the samples from different infection classes were roughly categorized into five groups, the concentrated distribution of sample points, particularly the significant overlap in the central region, resulted in insufficiently distinct differences in the spectral features among the various classes. This partial overlap was expected due to the gradual biochemical changes across *Verticillium dahliae* infection stages and factors such as biological variability in cotton stem composition, spectral similarity in cell wall components, and tissue heterogeneity, which can obscure class separation in PCA (Schulz and Baranska, 2007; Gierlinger and Schwanninger, 2006).This high degree of similarity complicates the ability of PCA to differentiate between the five VW infection classes, indicating that relying solely on PCA for feature extraction and classification has limited effectiveness, and should be further integrated with machine learning models for improved discrimination.

To further optimize the feature band selection, this study used two efficient feature selection algorithms: SPA and CARS. Both algorithms significantly reduced the dimensionality of the spectral data, achieving a reduction ratio of >81% (**Figure 5B**). Specifically, the SPA algorithm identified 84–98 feature bands, with a dimensionality reduction ratio of >82%. In contrast, the CARS algorithm also achieved a dimensionality reduction ratio of >81%. Notably, when applied to spectral data after the PolyFit baseline correction and SG smoothing, the CARS algorithm selected 104 feature bands. However, when applied to the spectral data after airPLS baseline correction and MSC processing, only seven feature bands were identified. These results demonstrate that both the SPA and CARS algorithms effectively extracted key features from spectral data while significantly reducing dimensionality, thereby providing efficient feature inputs for the subsequent construction of classification models.

To comprehensively evaluate the performance of various feature band selection methods, this study constructed an SVM-based classification model. The feature selection results from the SPA and CARS algorithms were inputted into the model for comparison. In addition, the classification effects of the eight significant Raman feature peaks obtained through inverse convolution calculations in Section 3.2 were also compared. The results indicate that the classification model based on the eight known feature bands was significantly less accurate for the training set than the results from the SPA and CARS algorithms (**Table 2**). This phenomenon suggests that although these feature peaks are prominent in the spectra, they do not provide sufficient information to fully reflect the biochemical characteristics of the samples, leading to limited classification performance. By contrast, the SPA and CARS algorithms extracted deeper insights from spectral data and identified more representative and discriminative feature bands, thereby significantly enhancing the accuracy of the classification model. In summary, although PCA has some value in the initial exploration of spectral data distributions, its classification effectiveness is limited. Conversely, the SPA and CARS algorithms substantially improved the performance of the classification model through efficient dimensionality reduction and feature extraction. Combined with the comparative results of the SVM models, this study confirmed the superiority of feature band selection algorithms based on chemometric approaches for classifying cotton stem Verticillium wilt, thereby providing an important methodological reference for future research.

**Table 2:** Classification accuracy of different feature band selection algorithms in SVM models.

Preprocessing Methods	Eight characteristic peaks		CARS		SPA	
	Train	Vaild	Train	Vaild	Train	Vaild
airPLS	0.65	0.62	0.70	0.68	0.72	0.70
SG-airPLS	0.68	0.65	0.73	0.70	0.75	0.73
SG-airPLS-(1/SG)′	0.70	0.68	0.75	0.72	0.78	0.75
SG-airPLS-MSC	0.55	0.52	0.60	0.58	0.63	0.60
SG-airPLS-SNV	0.60	0.58	0.65	0.62	0.68	0.65
SG-airPLS-SS	0.63	0.60	0.68	0.65	0.70	0.68

### 3.4 Cotton stem classification models for different disease levels

In this study, three distinct classification models, INFO-SVM, PSO-RF, and CSA-LSTM, were developed to classify cotton stem Verticillium wilt using Raman spectroscopy. Various preprocessing methods and feature band selection strategies were used for each model, and their performance was assessed using cross-validation and test sets. The effectiveness of each model in grading cotton stems with varying severities of VW is shown in **Table 3**.

**Table 3:** Results of each hierarchical model.

Preprocessing and Feature Selection Methods	INFO-SVM				PSO-RF				CSA-LSTM			
	Train		Vaild		Train		Vaild		Train		Vaild	
	Accuracy	F1 - score	Accuracy	F1 - score	Accuracy	F1 - score	Accuracy	F1 - score	Accuracy	F1 - score	Accuracy	F1 - score
R-CARS	0.963	0.963	0.900	0.911	0.938	0.936	0.600	0.627	0.688	0.656	0.600	0.541
R-SPA	0.625	0.604	0.500	0.330	0.663	0.664	0.200	0.157	0.650	0.634	0.500	0.363
airPLS-CARS	0.950	0.950	0.800	0.678	**0.975**	**0.975**	**0.700**	**0.544**	0.825	0.806	0.500	0.533
airPLS-SPA	0.625	0.616	0.400	0.294	0.963	0.963	0.400	0.394	0.638	0.602	0.400	0.360
SG-airPLS-CARS	0.588	0.575	0.800	0.738	0.838	0.820	0.700	0.747	0.725	0.717	0.500	0.448
SG-airPLS-SPA	0.650	0.641	0.800	0.633	0.813	0.805	0.600	0.493	0.700	0.682	0.500	0.430
**SG-airPLS-(1/SG)′-CARS**	**0.975**	**0.974**	**0.900**	**0.867**	0.963	0.962	0.800	0.638	0.738	0.726	0.600	0.574
SG-airPLS-(1/SG)′-SPA	0.675	0.667	0.700	0.544	0.963	0.965	0.500	0.367	0.700	0.651	0.400	0.367
SG-airPLS-MSC-CARS	0.388	0.235	0.200	0.133	0.948	0.949	0.600	0.471	0.513	0.468	0.700	0.748
SG-airPLS-MSC-SPA	0.813	0.814	0.800	0.811	0.963	0.961	0.400	0.360	0.638	0.632	0.600	0.550
SG-airPLS-SNV-CARS	0.913	0.912	0.500	0.385	0.925	0.926	0.500	0.440	**0.938**	**0.936**	**0.800**	**0.638**
SG-airPLS-SNV-SPA	0.925	0.924	0.500	0.600	0.950	0.948	0.700	0.611	0.588	0.512	0.200	0.137
SG-airPLS-SS-CARS	0.525	0.518	0.800	0.738	0.975	0.974	0.500	0.428	0.688	0.675	0.400	0.294
SG-airPLS-SS-SPA	0.800	0.775	0.700	0.643	0.963	0.963	0.600	0.653	0.750	0.705	0.400	0.380

Bold values indicate the best performance results under the corresponding algorithm.

**Figure 6:**
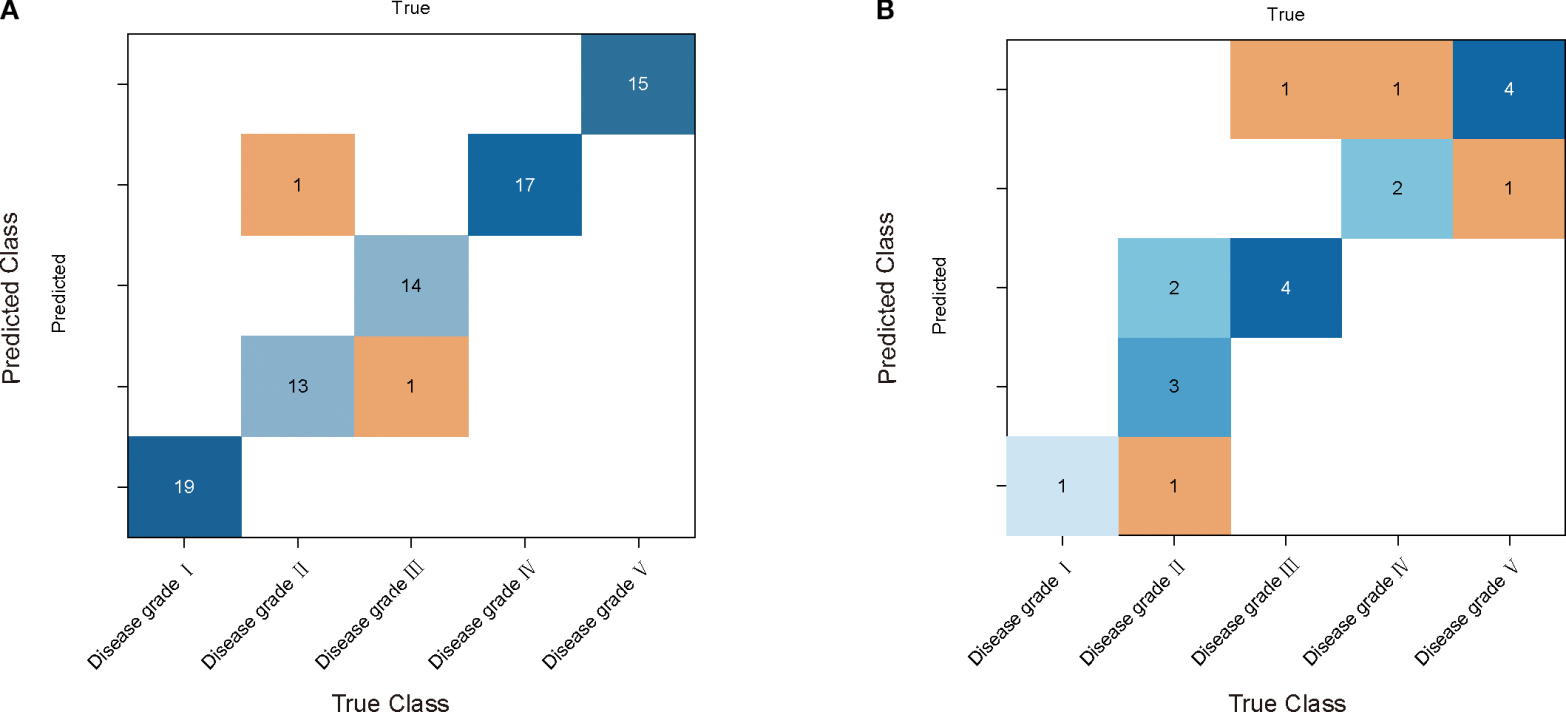
INFO-SVM cotton yellow wilt classification model results. **(A)** and **(B)** are the confusion matrices used by the model for the training and validation sets, respectively. The rows of each matrix represent the true categories and the columns represent the predicted categories.

The results of INFO-SVM modeling indicated that the classification model constructed using CARS for feature band selection was the most effective for grading cotton stems with varying severities of VW after SG-airPLS-(1/SG)′ processing. After ten-fold cross-validation and Monte Carlo sampling (100 times), the model achieved an accuracy of 97.5% and an F1-score of 0.974 on the modeling set. In contrast, the accuracy and F1-score for the validation set were 90.0% and 0.867, respectively (**Table 3**). The process of optimizing the spectral feature wavelengths is illustrated in **Figure 6A**. The optimal set of feature wavelengths was selected after 57 iterations, resulting in 58 feature wavelengths that accounted for 10.10% of the entire spectral band. These bands were identified as the optimal feature wavelength set when the RMSECV value was minimized. **Figure 6B** shows the confusion matrix of the INFO-SVM model for the training set. Its high accuracy and precision demonstrated the ability of the model to effectively differentiate between various sample classes. However, the performance of the validation set (**Figure 5C**) was slightly lower than that of the training set. The decrease in the F1-score suggests a slight reduction in the classification ability of the model on the validation set; nevertheless, the specificity remained high, indicating that the model performed well in identifying non-target classes. The performance of the test set is less different from that of the validation set, suggesting that the model exhibited good generalization capabilities for unseen data.

**Figure 7:**
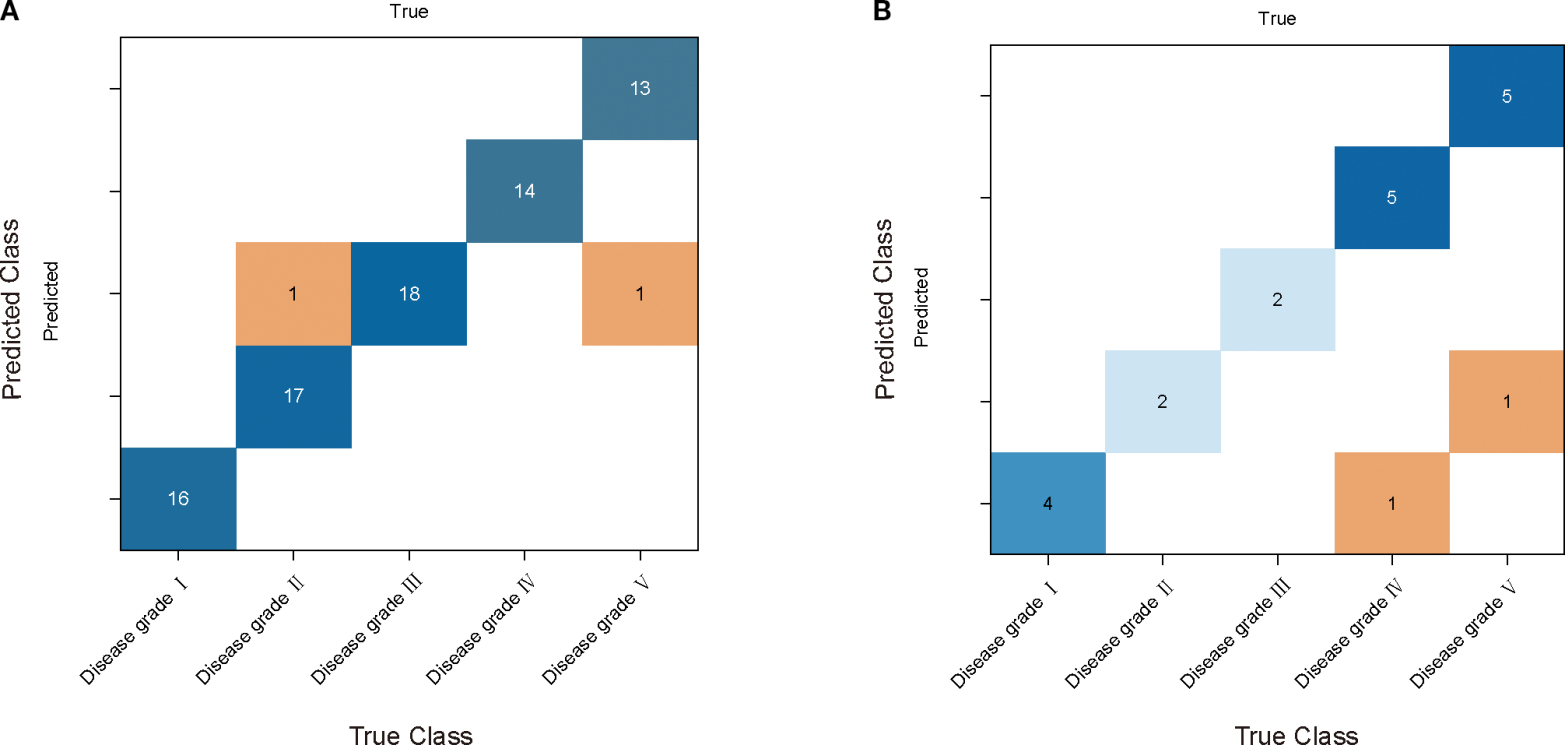
PSO - RF model performance comparison and analysis. **(A)** and **(B)** are its confusion matrices in the training and validation sets. The rows of each matrix represent the true categories and the columns represent the predicted categories.

The grading effectiveness of the PSO-RF model on cotton stems with varying severities of VW is illustrated in **Table 3**. The results indicated that after airPLS baseline correction, the PSO-RF model constructed using CARS for feature band selection demonstrated the highest efficacy in grading cotton stems with different levels of VW severity. The accuracy and F1-score for the modeling set were 0.975, whereas the accuracy and F1-score for the validation set were 70.0% and 0.544, respectively. The model used airPLS solely for baseline correction of the raw spectral data, with CARS utilized for feature band selection, as shown in **Figure 5D**. A total of 49 feature bands were identified, all of which fall within the Raman wavenumber range of 400 to 1800 cm^-^¹. This range encompasses the majority of characteristic peaks associated with lignin, proteins, and nucleic acids in cotton stems. **Figure 7** illustrate the confusion matrix for the PSO-RF hierarchical model applied to the training and validation sets. The grading effects of cotton stems with varying severities of cotton stem Verticillium wilt demonstrated different performance levels. The confusion matrix revealed that, in the training set, the sample prediction accuracies were consistently high, exceeding 90%. In contrast, the prediction accuracies in the validation set showed significant deviations, likely due to the limited number of samples.

**Figure 8:**
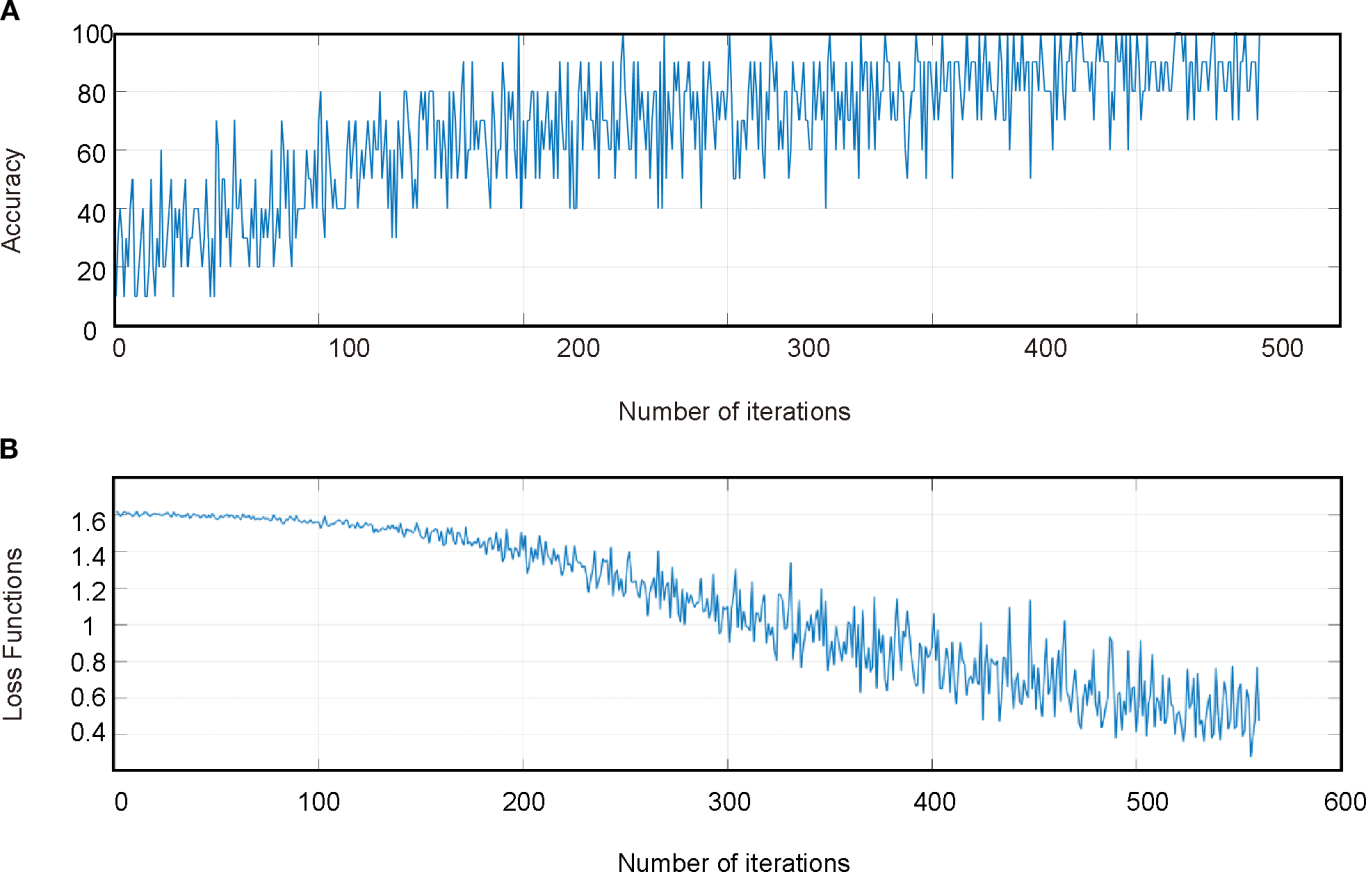
CSA-LSTM model training set process. **(A)** Accuracy iteration curves for the training set of the model. **(B)** Loss function curve for the training set of the model.

The results of the CSA-LSTM classification model indicated that after SG-airPLS-SNV processing, the model constructed using CARS for feature band selection achieved the best classification performance. It recorded an accuracy of 93.8% and an F1-score of 0.936 for the modeling set, whereas the validation set yielded an accuracy of 80.0% and an F1-Score of 0.638 (**Table 3**). Although the model demonstrated high accuracy on the modeling set, the relatively low F1-score on the validation set suggests that the model may have experienced some degree of overfitting during the validation phase. **Figure 8** illustrate the accuracy of the iteration and loss function curves for the CSA-LSTM model. As the number of iterations increased, the training set accuracy gradually improved and stabilized, whereas the loss function value decreased and converged to a lower value. However, this optimization may be overly reliant on the data features of the modeling set, which could diminish the generalization ability of the model when applied to the validation set data, ultimately affecting the F1-score of the validation set.

The results of this study demonstrated that an effective preprocessing method can enhance the accuracy of the classification detection model by as much as 97.5% on the training set. When evaluating the classification performance of the three models, the INFO-SVM model exhibited high accuracy and an F1 score on both the modeling and validation sets, surpassing those of the other two models on the validation set. The PSO-RF model performed well on the training set; however, its performance on the validation set declined significantly, indicating a weak generalization ability. In contrast, the CSA-LSTM model showed strong performance on the modeling set but had a low F1 score on the validation set, suggesting potential overfitting. Overall, the results indicate that the INFO-SVM model after SG-airPLS-(1/SG)′ -CARS preprocessing was the most effective for classifying and recognizing the Raman spectral data of cotton stems with varying levels of VW infection.

## 4 Discussion

The infestation of cotton with *V. dahliae* leads to significant alterations in various compounds within the stem, which can be effectively monitored using Raman spectroscopy. The analysis revealed that the intensity of the characteristic lignin peak at 1594 cm^-^¹ exhibited a dynamic pattern of an initial increase followed by a decrease. This phenomenon intuitively reflects the adjustment of plant defense strategies in response to VW. In the early stages of infestation, lignin accumulates in the cell wall as plants form a physical barrier against pathogenic fungi. However, as the disease progresses, the pathogen gradually degrades the lignin structure of the plant cell wall, resulting in a reduction in the lignin content and subsequent weakening of the intensity of the characteristic peaks (Pomar et al., 2004; Tian et al., 2023). This change was corroborated by the dynamic response of the characteristic peak at 931 cm^-^¹, which together illustrated the failure of plant defense mechanisms under sustained pathogen infestation. In addition, pectin degradation was reflected in the Raman spectra, with the intensity of the characteristic peak at 899 cm^-^¹ diminishing and shifting toward lower wavenumbers. This shift directly indicated the role of pectinase and further confirmed the synergistic degradation strategy used by pathogenic fungi for multiple components of the plant cell wall. This study not only verified the reliability and accuracy of Raman spectroscopy in phytopathological research but also demonstrated its unique advantage in elucidating the mechanisms of plant-pathogen interactions. Furthermore, a database of the Raman spectra of cotton stems was established, providing valuable data resources for future studies and facilitating the exploration of broader application scenarios.

Raman spectroscopy offers significant advantages in the analysis of biological samples, however, its spectral data are often influenced by fluorescence background, baseline drift, and noise interference (Schulz and Baranska, 2007; Smith and Dent, 2005). Consequently, spectral preprocessing is a crucial step in enhancing modeling effectiveness (Zhao et al., 2007). In this study, we systematically compared multiple spectral preprocessing methods and two baseline correction algorithms to optimize the quality of the spectral data for classifying cotton stem Verticillium wilt. The results indicate that the airPLS baseline correction effectively fitted the complex baselines and separated the target Raman signal using adaptive iterative weighted least squares. This approach significantly improves the SNR, outperforms traditional PolyFit for managing nonlinear baselines, and is particularly suitable for biological samples with strong fluorescence interference (Zhang et al., 2010). Spectral quality was further enhanced by combining SG smoothing with (1/SG)′, SG smoothing reduced random noise, whereas (1/SG)′ improved the distinction of feature peaks, particularly in weak-signal regions (Clupek et al., 2007). The comparison indicates that the SG-airPLS baseline correction combined with (1/SG)′ performed the best in enhancing the spectral quality and model classification performance. This approach significantly reduced the background fluorescence intensity and provided high-quality data support for subsequent feature extraction and machine-learning modeling. In this study, the SG-airPLS-(1/SG)′ combination strategy was applied for the first time to rapidly grade VW-affected cotton stems, thereby offering a new technical tool for the early diagnosis of agricultural diseases. Future research should explore the optimization of this combination with other pretreatment techniques and assess their generalizability for diagnosing other crop diseases, thereby providing broader technical support for disease management in agricultural production.

The intelligent screening mechanism of the feature bands exerts a dual driving effect on the performance optimization of the cotton stem Verticillium wilt classification model. In a comparison of downscaling methodologies, CARS demonstrated a parsing capability that surpassed traditional methods while effectively eliminating the most redundant noise bands and covariance interference in spectral data (Li et al., 2014). Compared with the eight intuitively selected Raman peaks, CARS extracted a greater number of feature bands and provided more comprehensive information, significantly enhancing the performance of the classification model, suggesting that an in-depth exploration of spectral potential information is crucial for model optimization. In contrast to PCA, which is hindered by the issue of pathological spectral feature aliasing due to linear decomposition (Tariq et al., 2024) and the risk of overfitting during band-independence screening with SPA, CARS constructs biologically interpretable feature subsets through dynamic integration of Monte Carlo sampling and partial least squares regression coefficients. Principal Component Analysis (PCA) often exhibit class overlap when analyzing Raman spectra of plant tissues due to spectral similarities and biological variability, limiting their ability to distinguish *Verticillium dahliae* disease severity classes (Gierlinger and Schwanninger, 2006; Egging et al., 2018). Meanwhile, the experimental results indicated that the F1-score of the INFO-SVM model, developed from 37 eigenbands screened by CARS, reached 0.974, representing a 6.82% improvement over the SPA method. This demonstrates the unique advantage of CARS in resolving nonlinear interactions between bands. Furthermore, CARS effectively enhanced the key biochemical response bands, particularly within the Raman shift interval of 1380 cm^-^¹ (the characteristic peak of Fusaric acid) and other specific markers for VW (Egging et al., 2018; Rosado et al., 2016), thereby providing a reliable spectral fingerprint library for the *in situ* detection of disease metabolites.

When constructing a rapid grading model for cotton stem Verticillium wilt, the INFO-SVM model demonstrated high accuracy, outperforming the PSO-RF and CSA-LSTM models. Its effectiveness in grading the detection of cotton stem Verticillium wilt was confirmed. The optimization benefits of the INFO algorithm stem from its global adaptive search capability for SVM hyperparameters, which effectively mitigates the limitations of traditional optimization methods that are often trapped in local optima (Li et al., 2015). This was achieved by introducing a nonlinear dynamic weighting strategy that reduced the sensitivity of the model to the initial parameters (Wan et al., 2024). Furthermore, the spectral data preprocessed by SG-airPLS-(1/SG)′ -CARS, when combined with the INFO-SVM model, exhibited high specificity and an F1-score of 0.867 on the validation set, confirming its ability to distinguish between different infection classes of VW in a complex noise environment. Although there was a slight decrease in the F1-score compared with the training set, the overall generalization performance of the model remained stable, indicating the feasibility of the method for grading the detection of other crop diseases.

Despite the promising results achieved in this study, several limitations should be acknowledged to guide future research. First, the current dataset, though carefully curated, may lack sufficient representativeness across diverse cotton cultivars, growth stages, and environmental conditions. Early-stage infection samples were particularly limited, which could affect the model’s sensitivity to initial symptom detection. Expanding the spectral database with longitudinal field samples is essential to enhance generalization. Second, while Raman spectroscopy offers high specificity, its performance in field applications is often compromised by environmental interferences, such as ambient light, temperature fluctuations, and humidity, which can introduce noise and baseline drift (Smith and Dent, 2005; Farber et al., 2019a). Developing robust preprocessing algorithms or noise-invariant deep learning architectures could improve adaptability to these real-world conditions. Third, although machine learning models delivered high accuracy, their “black-box” nature limits agronomic interpretability. In the future, the Shapley Additive exPlanations (SHAP) interpretable framework can be integrated into a feature band screening system. By quantifying the contributions of band weights, we can create spectral response-metabolic pathway correlation maps to further address the limitations of the traditional “black box” model. Lastly, the reliance on benchtop Raman systems restricts field deployability due to their high cost, large size, and susceptibility to fluorescence interference in biological samples (Farber et al., 2019a). Future investigations could explore the integration of alternative laser wavelengths, such as 785 nm and 1064 nm, which are more common in portable Raman systems. The 785 nm laser provides a strong balance between signal intensity and fluorescence suppression, while the 1064 nm laser significantly reduces fluorescence interference in biological samples, offering a particular advantage for in-field diagnosis of pigmented plant tissues (Smith and Dent, 2005). Exploring low-cost portable spectrometers coupled with lightweight models could facilitate scalable, on-farm diagnostics. Addressing these limitations will be critical for translating this technology into practical precision agriculture tools.

This study demonstrates a novel Raman spectroscopy-machine learning frame-work that enables early and accurate detection of Verticillium wilt (VW), a major advancement in the field of plant disease diagnosis. By leveraging the molecular specificity of Raman spectroscopy to identify pre-symptomatic biochemical changes and combining it with optimized machine learning algorithms, we achieve sensitive detection of early infection with up to 85% recall. This study offers unique advantages for early cotton yellow wilt surveillance, including minimal sample preparation, rapid analysis, and high classification performance. While the current results demonstrate the method’s good pre-symptomatic detection capability, future studies should extend the spectral database to include a more diverse range of early infection time courses and environmental conditions to enhance the robustness of the model. This early detection approach fundamentally shifts crop protection strategies from reactive treatment to preventive intervention, providing an important technological foundation for implementing precision agriculture systems that can identify and mitigate disease threats before visible symptoms appear. Further integration with portable spectroscopic equipment and an interpretable artificial intelligence framework will accelerate the translation of this technology into a practical early warning system for field applications.

## 5 Conclusion

In this study, hierarchical detection of VW on cotton stems was achieved using Raman spectroscopy in conjunction with machine learning algorithms. The combination of Raman spectroscopy and the CARS feature band selection algorithm effectively extracted the spectral features associated with VW, thereby significantly enhancing the accuracy of the classification model. By comparing the classification models constructed with various optimization algorithms, it was determined that the classification accuracy of the INFO-SVM model on the validation set reached 90%, outperforming the PSO-RF (70%) and CSA-LSTM (80%) models. This indicates that the INFO-SVM model is more suitable for Raman spectral grading detection of cotton stem Verticillium wilt. This method established a rapid and accurate disease classification model, providing a novel approach for the early detection of cotton stem Verticillium wilt. It offers the advantages of high efficiency and low cost and delivers reliable data support for subsequent research. This study confirmed the significant potential of combining Raman spectroscopy with machine learning for diagnosing agricultural diseases, thereby offering technical support for intelligent disease monitoring and management. In the future, this method can be further disseminated and applied for the early diagnosis of other crop diseases, thereby promoting the intelligent development of agricultural disease-monitoring technology.

## Data Availability

The datasets presented in this study can be found in online repositories. The names of the repository/repositories and accession numbers can be found in the article/supplementary material.
